# How can medical schools combat declining mental health amongst their students during the COVID-19 quarantine period?

**DOI:** 10.12669/pjms.37.3.4321

**Published:** 2021

**Authors:** Kajani Subhaskaran, Francesca N Young

**Affiliations:** 1 Miss Kajani Subhaskaran, Bsc (Hons), Warwick Medical School, Medical School Building, Coventry, CV4 7AL, United Kingdom; 2 Miss. Francesca Naomi Young, Bsc (Hons), Warwick Medical School, Medical School Building, Coventry, CV4 7AL, United Kingdom

To the Editor,

We have read the review ‘COVID-19 Pandemic: Impact of Quarantine on Medical Students’ Mental Wellbeing and Learning Behaviours’ by Meo and colleagues.^1^ We would like to make some contributions to this insightful report.

This study found that 44% of medical students felt emotionally detached from their family and fellow students during quarantine and 39% experienced anxiety. We are grateful to Meo et al.^1^ for highlighting the need for interventions which target medical students’ mental wellbeing during quarantine. Based on these findings, we would like to contribute strategies which could be incorporated into the curriculum in order to help students adapt to such circumstances.

Kazerooni et al.^2^ reported the success of a social media platform set up at Shiraz University’s medical school whereby senior students were trained to mentor junior students on strategies for coping with anxiety and maintaining contact with others. This was found to be beneficial for both parties as 71% of junior students reported that the platform was a significant tool in helping them adjust to the unprecedented circumstances, whilst senior students found that it promoted professional growth required to be competent physicians. It would have been valuable for Meo et al.^1^ to also survey the students regarding what, if any, provisions had been implemented by the university to help students during quarantine.

Mindfulness-based interventions in medical education have become increasingly popular over time. A recent study^3^ reported that an online university mindfulness course for health profession students was highly successful during the COVID-19 pandemic. All students found that practising mindfulness techniques enabled them to cope with anxieties during the quarantine period. The course also allowed students to feel more connected and appreciative of their own personal relationships.

By implementing mindfulness teaching into medical school curriculums and creating platforms for peer support, there will be more resources available to support students’ mental health during quarantine.

## Authors’ Contribution:

**FNY and KS** did the writing & editing of this manuscript.
